# Impact of Marasmic Malnutrition on Visceral Leishmaniasis: Progression and Treatment Efficacy in a Murine Model

**DOI:** 10.3390/nu17050849

**Published:** 2025-02-28

**Authors:** Taiana Ferreira-Paes, Luiza F. O. Gervazoni, Paula Seixas-Costa, Paula Mello De Luca, Elmo Eduardo Almeida-Amaral

**Affiliations:** 1 Laboratório de Bioquímica de Tripanosomatídeos, Instituto Oswaldo Cruz, Fundação Oswaldo Cruz, Rio de Janeiro 21040-360, Brazil; taiana.paes@ioc.fiocruz.br (T.F.-P.); luiza.gervazoni@ioc.fiocruz.br (L.F.O.G.); paulascosta2@yahoo.com.br (P.S.-C.); 2 Laboratório de Imunoparasitologia Clínica, Instituto Oswaldo Cruz, Fundação Oswaldo Cruz, Rio de Janeiro 21040-360, Brazil; pmdeluca@ioc.fiocruz.br

**Keywords:** marasmic malnutrition, visceral leishmaniasis, host immune status, treatment, leishmania infantum infection, refeeding intervention

## Abstract

Background/Objectives: Malnutrition and visceral leishmaniasis are major public health problems that are responsible for millions of deaths across many countries. Leishmaniasis development and progression are associated with the host immune status. In this context, malnutrition can directly affect the course of leishmaniasis, impairing several components of the immune system. Moreover, malnutrition directly interferes with the tropism of *Leishmania* in organs, affecting host susceptibility. Therefore, this work aimed to evaluate the influence of nutritional status on the establishment, progression, and treatment of *Leishmania infantum* infection in malnourished and refed mice. Methods: BALB/c mice were fed either a control or restricted diet, infected with *L. infantum* promastigotes, and treated with meglumine antimoniate, the standard drug for treating visceral leishmaniasis. The effects of infection were evaluated through limiting dilution analysis (LDA). Results: Compared with control mice, malnourished and refed mice presented a lower parasitic load in the spleen, which correlated with spleen atrophy, and the refeeding process partially reversed but did not fully rescue the infection status. Both groups presented a high parasitic load in the liver. Marasmic malnutrition appeared to impair the efficacy of leishmaniasis treatment; however, the refed groups exhibited a robust decrease in the parasite load, which was comparable to that in the control group subjected to treatment. Conclusions: Our data suggested that marasmic malnutrition affects the establishment and progression of *Leishmania* infection, in addition to reducing the efficacy of standard treatment. Furthermore, the refeeding intervention used did not fully reverse the observed effects. These findings highlight the potential importance of nutritional interventions in the clinical management of visceral leishmaniasis in malnourished populations.

## 1. Introduction

The nutritional status of the host has a direct effect on the modulation of immunity and susceptibility to infectious and parasitic diseases [1,2]. The presence of these infections can lead to a reduction in the host’s ability to absorb nutrients, which can result in malnutrition and establish a self-perpetuating cycle [3,4,5].

According to the World Health Organization (WHO), malnutrition is characterized by either an inadequate or an excessive intake of nutrients or energy, leading to underweight, overweight, or obesity [6,7]. In 2021, the Food and Agriculture Organization (FAO) of the United Nations reported that the global prevalence of undernourishment affected an estimated 767.9 million people [8].

Leishmaniasis comprises a group of neglected tropical diseases caused by protozoan parasites of the genus *Leishmania*. While all neglected tropical diseases are reported to affect 149 countries worldwide, leishmaniasis by itself is endemic in 98 countries/territories. Currently, 700 thousand to 1 million new cases of leishmaniasis are estimated to occur per year, and approximately 1 billion people worldwide are vulnerable to infection [9].

Leishmaniasis presents in two main clinical forms: cutaneous and visceral. Cutaneous leishmaniasis manifests through skin lesions, which can be single and painless, multiple, and disseminated, or it causes destructive lesions on both the skin and mucous membranes. In contrast, visceral leishmaniasis affects internal organs, leading to hepatomegaly, splenomegaly, fever, anemia, weight loss, and bone marrow damage [9,10].

Additionally, visceral leishmaniasis is the most lethal form of the disease when left untreated or inadequately treated, and it ranks as the second deadliest disease in tropical and subtropical regions. Globally, seven countries, including Brazil, Ethiopia, India, Kenya, Somalia, Sudan, and South Sudan, account for 90% of the world’s visceral leishmaniasis cases [1,9,10]. Brazil, in particular, has the highest number of cases in the Americas, representing 97% of reported cases [11].

Several studies have highlighted the age dependence of visceral leishmaniasis susceptibility, showing that it tends to preferentially affect individuals with weaker immune systems, such as children and elderly individuals [1,2,9,10,11,12]. However, the host immune system is not the only factor associated with the development and progression of visceral leishmaniasis. Socioeconomic conditions, migration from rural to urban areas, environmental changes, immunosuppressive diseases, coinfections, and malnutrition can also influence the course of this disease [13,14].

Undernutrition has been widely recognized as a significant risk factor for the progression and severity of visceral leishmaniasis [15]. Prospective studies in children who developed visceral leishmaniasis have shown that those with moderate to severe undernutrition before infection had an 8.7-fold increased risk of developing classic and severe forms of the disease compared with that in well-nourished children, who remained in the subclinical phase [16]. Additional research has indicated that undernutrition not only heightens susceptibility to more severe forms of visceral leishmaniasis but also accelerates the overall disease progression [17,18,19]. A study analyzing 29,570 consecutive visceral leishmaniasis cases from Brazil, East Africa, Nepal, and India between 1997 and 2009 reported high rates of malnutrition among patients under five years of age, with the prevalence reaching 39.3% in the Upper Nile region of southern Sudan [20].

The interplay between undernutrition and visceral leishmaniasis has also been investigated in experimental murine models. Research has established an undernutrition scale for mice on the basis of human anthropometric classification, which has shown that undernutrition impairs the barrier function of lymph nodes in *L. donovani*-infected mice. This disruption facilitates earlier visceralization of the parasite, likely due to the depletion of phagocytic cells within the lymph nodes [21,22]. Moreover, undernourished mice presented a reduction in the number of skin-resident dendritic cells migrating to satellite lymph nodes, along with dysregulated expression of chemokines and receptors essential for dendritic cell migration [23].

Marasmus, a form of protein–energy malnutrition, is one of the most common and severe types of malnutrition and is caused by inadequate protein and calorie intake and a resultant deficiency [24,25]. This condition causes a decline in physical strength, making individuals, especially children, particularly vulnerable to infectious diseases [21].

Although some studies have demonstrated the relationship between malnutrition and visceral leishmaniasis in a murine model [12,22,26,27,28,29], no studies have specifically examined whether marasmic-type malnutrition is associated with visceral leishmaniasis.

Therefore, this study aimed to evaluate the impact of marasmic malnutrition on the development, progression, and treatment of visceral leishmaniasis. Given the lack of studies on marasmic malnutrition using mouse models and their importance in disease studies, a mouse-feeding protocol was recently developed and validated to mimic marasmus [30]. Marasmic malnutrition influences the establishment and progression of *Leishmania* infection, as well as the efficacy of standard treatment, whereas refeeding intervention completely reverses these effects. These findings highlight the potential importance of nutritional interventions in the clinical management of visceral leishmaniasis in malnourished populations.

## 2. Materials and Methods

### 2.1. Parasites

*L. infantum* (MHOM/MA/67/ITMAP263) promastigotes were cultured at 26 °C in Schneider’s insect medium (Sigma Aldrich, St. Louis, MO, USA) supplemented with 20% fetal calf serum (FCS), 100 U/mL penicillin G potassium, and 100 μg/mL streptomycin at pH 6.9. For in vivo infection, promastigotes cultured for a maximum of 5 passages and freshly recovered from infected BALB/c mice were used.

### 2.2. Mice

Weanling female BALB/c mice at age 3 weeks were acquired from Instituto de Ciência e Tecnologia em Biomodelos (ICTB—FIOCRUZ, Rio de Janeiro, Brazil) and kept in specific pathogen-free conditions. The Ethics Committee on the Use of Animals at Instituto Oswaldo Cruz reviewed and approved all procedures involving the animals (license L-011/2017).

### 2.3. Diets

Chow was purchased from PragSoluções Biociências (Jaú, SP, Brazil) and administered according to the protocol outlined by [30] Ferreira-Paes et al. (2021). The control diet consisted of 24.18% crude protein, 65.93% carbohydrates, and 9.89% ethereal extract (fat), providing 15.24 kJ/100 g, and the restricted diet contained 8.79% crude protein, 36.26% carbohydrates, and 4.95% ethereal extract (fat), providing 7.62 kJ/100 g. Both diets were supplemented with fiber and included all essential micronutrients necessary for the survival of the mice.

### 2.4. Feeding Protocol, Infection, and Experimental Procedure

Initially, BALB/c mice were acclimated for one week with a control diet (30 g/day). Afterward, the mice were randomly divided into six groups. The control group (C) was fed the control diet at 30 g per day. The malnourished (M) and refed (R) groups were given a restrictive diet of 30 g per day. On day 3, all the mice were intraperitoneally infected with stationary-phase *Leishmania infantum* promastigotes (1 × 10^8^ promastigotes/100 µL). Starting on day 10 (7 days post-infection), the mice were treated intramuscularly with either 100 mg/kg/day of meglumine antimoniate diluted in saline solution (final volume: 100 µL) or 100 µL of saline solution administered into the pad of the right hind paw for 6 consecutive days. On day 10, the refeeding groups were also switched to the control diet (30 g/day) until day 16, during which they underwent the refeeding phase. On day 16 (13 days post-infection), the mice were euthanized. This experiment was performed in three independent trials, with a total of 15 animals per group. For each trial, 5 mice were housed per cage, with unrestricted access to water.

### 2.5. Phenotypic Parameters

For the assessment of nutritional status, the mice were weighed daily via a calibrated digital scale. The initial average body weight at the start of the experiment was 20 g. After the 16-day experimental period, the percentage weight gain was calculated. For the control and malnourished groups, the final body weight on day 16 was divided by the initial body weight on day 0. In the refeeding groups, two separate calculations were performed: the body weight on the last day before refeeding (day 10) was divided by the initial weight on day 0, and the final body weight on day 16 was divided by the weight recorded immediately before refeeding (day 11).

### 2.6. Evaluation of Parasite Load by Limiting Dilution Analysis (LDA)

Parasite loads in the spleen and liver were determined via quantitative limiting dilution analysis (LDA). After euthanasia, the organs were collected, weighed, and macerated in Schneider’s medium supplemented with 20% fetal bovine serum (FBS). The cell suspensions were then placed in 96-well plates containing Schneider’s medium and serially diluted. The plates were incubated at 26 °C for 7 days, and the wells were examined daily via an inverted microscope. The number of parasites/mg of tissue was estimated on the basis of the total weight of the collected tissue and the parasite load in the serial dilution, as described.

### 2.7. Statistical Analysis

Mean and standard deviations were derived from a minimum of three independent experiments. Statistical analyses were conducted via GraphPad Prism 7 (GraphPad Software, La Jolla, CA, USA). An analysis of variance (ANOVA) or *t* test was employed, followed by post hoc testing via Tukey’s or Mann–Whitney methods, as appropriate. Statistical significance was determined at a threshold of *p* < 0.05.

## 3. Results

**Figure 1:**
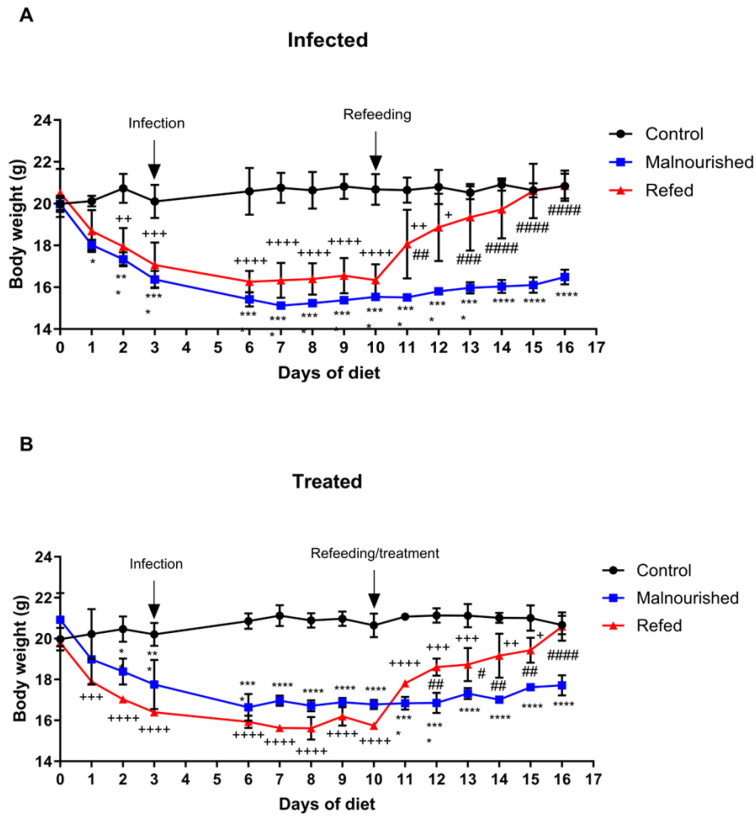
The weights of BALB/c mice subjected or not subjected to experimental malnutrition and refeeding, infected with *L. infantum,* and treated with meglumine antimoniate were evaluated. Body weights of infected (**A**) and treated (**B**) mice. Black dots: control group, blue dots: malnourished group, red dots: refed group. Statistical analysis: *^,^ +, # *p* ≤ 0.05, **, ++, ## *p* ≤ 0.009, ***, +++, ### *p* ≤ 0.0009, ****, ++++, #### *p* < 0.0001. (*) malnourished vs. control, (+) refed vs. control, (#) refed vs. malnourished. The values represent the means ± standard errors of three independent experiments with 5 animals per group. Two-way ANOVA (body weight) and one-way ANOVA with Tukey’s post hoc test (weight gain) were used for analysis.

To demonstrate the effects of marasmic malnutrition on the establishment, progression, and effectiveness of standard visceral leishmaniasis treatment, the initial objective was to determine whether infection and treatment impacted the weight of control, malnourished, and refed infected mice. Neither infection nor treatment significantly affected the weight of the mice evaluated (Figure 1A,B). During infection, the untreated malnourished mice did not lose body weight (16.37 g on day 3 and 16.49 g on day 16), whereas the refed infected mice (before refeeding; days 3 to 10) lost only approximately 4% of their body weight. In contrast, the control infected mice gained approximately 5% of their body weight. Furthermore, after refeeding, the refed infected mice and the refed treated mice recovered from their weight loss, gaining approximately 22% of their body weight and reaching weights similar to those of the control infected and control treated mice.

**Figure 2:**
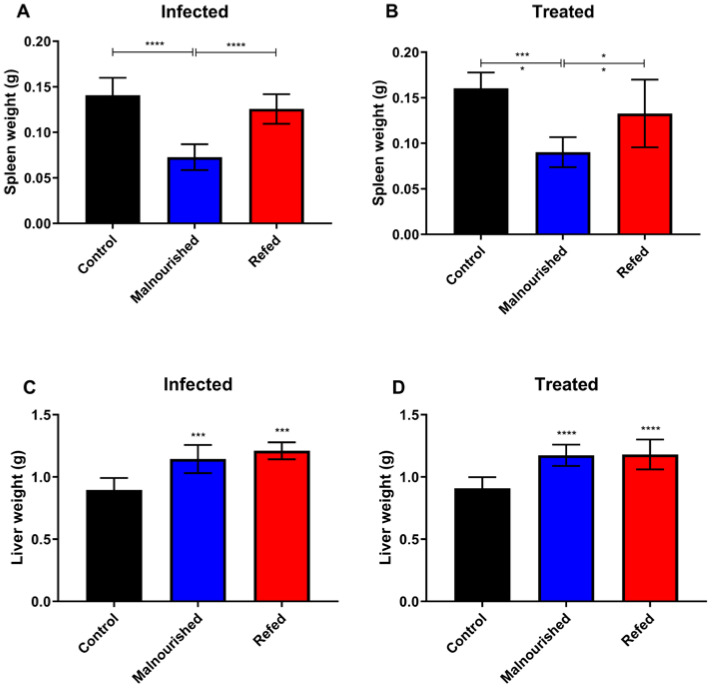
Organ weights of BALB/c mice subjected or not subjected to experimental malnutrition and refeeding, infected with *L. infantum,* and treated with meglumine antimoniate. Spleen weight (**A**,**B**) and liver weight (**C**,**D**). The mice were euthanized, and the spleen and liver were removed aseptically. The organs were weighed on a digital scale. Black: control group, blue: malnourished group, red: refed group. * *p* ≤ 0.05, *** *p* ≤ 0.0009, **** *p* ≤ 0.0001. (*) represents a difference from the control group. The values are presented as the means ± standard errors of 3 independent experiments with 15 animals per group. Student’s *t* test with the Mann–Whitney post hoc test was used for analysis.

Given the tropism of *Leishmania* species that cause visceral leishmaniasis in organs such as the liver and spleen, the impact of marasmic malnutrition on visceral leishmaniasis progression, treatment response, and effectiveness was assessed by analyzing organ weights. Compared with control and refed mice, malnourished mice presented significantly lower spleen weights, irrespective of their infection status (a 48% to 42% reduction in spleen weight; Figure 2A) or treatment (a 44% to 32% reduction in spleen weight; Figure 2B). By the conclusion of the experiment (day 16), the spleen weights of the refed mice were comparable to those of the control group.

Compared with those of the control mice, the livers of both the marasmic malnutrition and the refed mice were significantly enlarged in both the infected and treated groups (Figure 2C,D). In mice with marasmic malnutrition, the liver weight increased by 28% in the infected group and 29% in the treated groups compared with in the control group. Among the refed mice, the infected and treated groups presented 35% and 30% increases in liver weight, respectively, compared with that in the control group. No differences in liver weight were observed in the control group due to *L. infantum* infection or meglumine antimoniate treatment.

**Figure 3:**
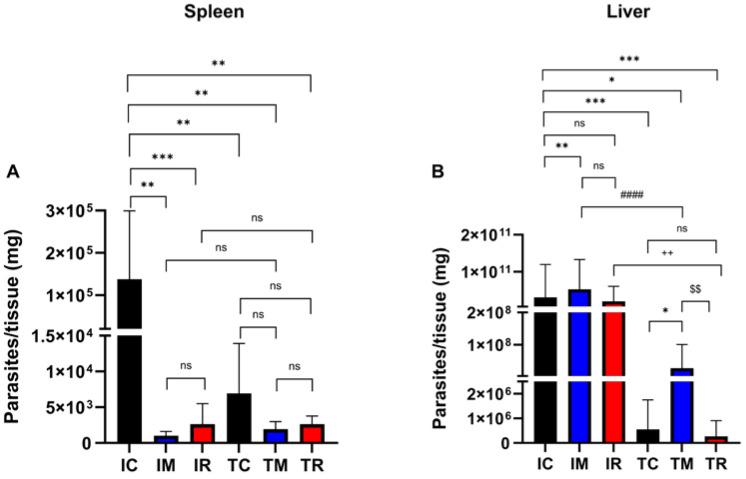
Evaluation of the parasitic load of BALB/c mice subjected or not subjected to experimental malnutrition, refeeding, and induction of visceral leishmaniasis. Quantification of parasites present in the spleen (**A**) and liver (**B**) of mice infected intraperitoneally with *L. infantum* (1 × 10^8^ cells/100 µL). After 7 days of infection, the mice were treated with or without meglumine antimoniate (100 mg/kg/day). At the end of the experiment, the mice were euthanized, and the spleen and liver were aseptically removed. The parasite load was determined via limiting dilution analysis (LDA). ++ *p* ≤ 0.0009, #### *p* ≤ 0.0001, ^$$^ *p* ≤ 0.009, * *p* ≤ 0.05, ** *p* ≤ 0.009, *** *p* ≤ 0.0009, ns = not statistically significant. The values are presented as the means ± standard errors of 3 independent experiments with 5 animals in each group. Student’s *t* test with the Mann–Whitney post hoc test was used for analysis. IC: infected control, IM: infected malnourished, IR: infected refed, TC: treated control, TM: treated malnourished, TR: treated refed.

The parasite load in the visceral leishmaniasis experimental mouse model is a key metric for understanding visceral leishmaniasis progression and treatment effectiveness. To evaluate the effects of malnutrition on *Leishmania* infection in vivo, BALB/c mice were infected with *L. infantum* promastigotes. At the end of the experiment, the spleens and livers were collected, and LDA assays were performed to determine the parasite load. In both organs, infection establishment and treatment efficacy were observed and compared between the infected and treated control groups. Compared with infection in the control group (IC: 1.37 × 10^5^ parasites/mg of tissue), marasmic malnutrition dramatically affected infection progression in the spleen of BALB/c mice (*p* = 0.0012), and the refeeding process did not create a favorable environment for *Leishmania* infection (*p* = 0.0007) (IM: 1.01 × 10^3^ and IR: 2.63 × 10^3^ parasites/mg of tissue; Figure 3A). While marasmic malnutrition impaired *Leishmania* infection in the spleen of BALB/c mice, it appeared to significantly promote infection in the liver of BALB/c mice compared with that in the infected controls (*p* = 0.0036) (IC: 3.0 × 10^10^ and IM: 5.25 × 10^10^ parasites/mg of tissue). However, the refeeding process restored the infection levels to those of the control group (*p* = 0.9514; IR: 1.94 × 10^10^ parasites/mg of tissue) (Figure 3B).

In the control group, meglumine antimoniate treatment resulted in a 10^2^-fold reduction in the parasite load in the spleen (95% inhibition; TC: 6.94 × 10^3^ parasites/mg of tissue) (Figure 3A) and a 10^5^-fold reduction in the parasite load in the liver (99.9% inhibition; TC: 5.5 × 10^5^ parasites/mg of tissue) (Figure 3B). However, the malnutrition protocol significantly affected the effectiveness of meglumine antimoniate treatment, leading to only a 10^3^-fold reduction in the liver parasite load (TM: 2.7 × 10^7^ parasites/mg of tissue), which was notably lower than that in the control group. Refeeding the BALB/c mice successfully restored the effectiveness of meglumine antimoniate treatment, resulting in a 10^5^-fold decrease in liver parasite load (TR: 2.7 × 10^5^ parasites/mg of tissue), comparable to that observed in the control group (Figure 3B). These findings emphasize the crucial impact of refeeding on visceral leishmaniasis treatment.

To assess the additional impacts of malnutrition, blood was collected from BALB/c mice for hematological and biochemical analysis. The malnourished mice presented a significant decrease in the number of leukocytes, indicating an impact on the immune system. However, the refeeding process appeared to return the levels to normal (Supplementary Table S1). Neither *Leishmania* infection nor meglumine antimoniate treatment altered this profile. Additionally, the malnourished mice presented a slight increase in serum cholesterol levels (Supplementary Table S2).

## 4. Discussion

The global burden of malnutrition is a serious and persistent health problem. According to the WHO, in 2022, 390 million adults were underweight, and half of the deaths of children under 5 years of age were linked to undernutrition [8,31]. Malnutrition is a major contributor to immunodeficiency, exerting profound effects on both innate and adaptive immune responses. Its impact compromises various immune functions, leading to increased susceptibility to infections and an impaired ability to mount effective immune defenses [4,5,32].

Individuals with malnutrition become susceptible to several infectious and parasitic diseases. Immunosuppression resulting from nutritional deficiency allows several microorganisms to become pathogenic, leading to numerous illnesses [33]. Multiple studies have shown associations between malnutrition and infections caused by different pathogens, including viruses [34,35], intestinal parasites [36,37], and protozoans [14,38].

Leishmaniasis is a complex of diseases caused by protozoan parasites of the genus *Leishmania*. Visceral leishmaniasis is the most serious clinical manifestation of leishmaniasis because of its high mortality rate if it is left untreated. Individuals with a weakened immune system are more susceptible to contracting visceral leishmaniasis and developing its most severe symptoms, and the nutritional status of the host plays a critical role in the disease progression and treatment outcomes [18,39,40].

Protein–energy malnutrition (PEM) and visceral leishmaniasis represent significant political, social, and public health challenges, as both diseases are closely linked to poverty and are responsible for millions of deaths worldwide. Malnutrition increases the sensitivity to infections such as leishmaniasis by impairing immune system functionality, leading to more severe clinical outcomes. Malnutrition, particularly in patients with visceral leishmaniasis, is directly correlated with poorer treatment outcomes. Patients with a compromised nutritional status have a lower response to therapy, emphasizing the need for comprehensive treatment approaches that address both disease and nutritional deficits. Furthermore, the critical role of host nutritional status in influencing the effectiveness of treatment suggests that malnutrition exacerbates the severity of infection and impairs immune responses essential for recovery [2,8]. Therefore, a vicious cycle exists between malnutrition and infection: infections can cause loss of appetite, indirectly leading to inadequate food intake, which in turn worsens the malnutrition status and increases individuals’ vulnerability to subsequent infections [41].

As previously reported, malnutrition is a key risk factor for the development and progression of visceral leishmaniasis [42]. However, there is limited information in the literature regarding the impact of marasmic malnutrition on *Leishmania infantum* infection, particularly concerning the effectiveness of leishmaniasis treatment. Therefore, understanding the relationships between marasmic malnutrition and the development, progression, and treatment of visceral leishmaniasis is crucial.

Mice with marasmic malnutrition in both the infected group and the treated group showed a reduction in splenic weight. However, the spleen weights were restored in the refed mice. In contrast, both the malnourished and refed mice presented increased liver weights. Studies have shown that *Leishmania infantum*-infected BALB/c mice fed a low-protein isocaloric diet presented decreased spleen weight and increased liver weight. These findings indicate that protein malnutrition leads to a significant reduction in spleen weight, reflecting impaired immune function, whereas the increase in liver weight in response to infection suggests a complex interaction between malnutrition and the body’s response to *Leishmania* infection [43].

An assessment of the impact of marasmic malnutrition on visceral leishmaniasis revealed that malnourished mice had a lower parasite load in the spleen. Notably, the refeeding protocol did not fully reverse the *Leishmania* infection in the refed group, as the infection burden in the spleen remained reduced. However, in the liver, an increased parasite load was observed in the infected malnourished mice, and the refeeding process restored the parasite levels to those observed in the control group. These results are consistent with findings from He et al. (2024), who reported a greater parasite load in the livers of undernourished mice infected with *Leishmania donovani* [12].

Our findings indicate that *Leishmania* parasites exhibited a greater preference for the liver than for the spleen in mice with marasmic malnutrition. This result leads to two possible hypotheses: (1) Malnourished mice exhibit a reduced spleen size, decreased cell count, and gradual depletion of the lymphocyte population [44]. In the present study, atrophy of the spleen was observed in mice with marasmic malnutrition, suggesting that parasites may struggle to establish infection in this organ due to impaired immune function. (2) Malnutrition can lead to fat accumulation in the liver, causing hepatic steatosis [31,45,46,47]. Since *Leishmania* amastigotes utilize fatty acids as an energy source [48] and the compromised splenic environment hinders infection establishment, this may explain the parasite’s preference for the liver under malnutrition conditions. The abundant nutritional supply in the liver may allow *Leishmania* to sustain itself in the host and perpetuate the infection. As a result, the parasite load is reduced in the spleen, whereas the susceptibility to infection increases in the liver, indicating potential parasitic adaptation to adverse nutritional conditions. However, further studies are needed to better understand this phenomenon.

The relationship between marasmic malnutrition and visceral leishmaniasis treatment has been poorly explored in the literature. In this context, a study was conducted to assess the efficacy of meglumine antimoniate treatment in malnourished and refed mice. Mice with marasmic malnutrition presented a decrease in the parasitic load in the liver, although this reduction was less pronounced than that in the treated control group. These findings suggest that malnutrition may reduce the effectiveness of leishmaniasis treatment. Conversely, renourished (refed) mice presented a substantial reduction in parasitic load, similar to that observed in the control mice, indicating that, after refeeding, the treatment regained its full effectiveness.

Visceral leishmaniasis is often linked with malnutrition, which can exacerbate the severity of the infection and serve as a major risk factor for poorer clinical outcomes and treatment responses. Notably, the treatment of visceral leishmaniasis is determined on the basis of the patient’s weight [6,26,39].

During the progression of visceral leishmaniasis, several changes, such as thrombocytopenia, leukopenia, neutropenia, hypoalbuminemia, anemia, and elevated liver enzyme levels, are commonly observed [42,48]. However, the precise mechanisms underlying the complex interaction between visceral leishmaniasis and malnutrition, including how malnutrition impacts the immune system and disease progression, are not fully understood and require further investigation.

## 5. Conclusions

In conclusion, the present study provides valuable insights into the complex interplay between marasmus and visceral leishmaniasis, showing that malnutrition markedly affects both the course of infection and the treatment outcome following meglumine antimoniate administration. These findings underscore the potential role of nutritional interventions in the clinical management of visceral leishmaniasis, particularly in populations at risk of malnutrition. Further research is needed to elucidate the underlying mechanisms of these interactions and to develop therapeutic strategies.

## Data Availability

The original contributions presented in the study are included in the article/Supplementary Material, further inquiries can be directed to the corresponding author.

## Supplementary Materials

The following supporting information can be downloaded at: https://www.mdpi.com/article/10.3390/nu17050849/s1, Table S1: Hematological Parameters of Infected Control, Malnourished, and Refed Mice, Treated or Untreated; Table S2: Biochemical Parameters of Infected Control, Malnourished, and Refed Mice, Treated or Untreated.
