# Supplementary material for: Impact of Marasmic Malnutrition on Visceral Leishmaniasis: Progression and Treatment Efficacy in a Murine Model
Source: Nutrients. 2025 Feb 28;17(5):849. doi: 10.3390/nu17050849 (PMC11901435; doi:10.3390/nu17050849)
Supplement: Supplementary file 1 [file nutrients-17-00849-s001.zip › nutrients-3237709-supplementary.pdf]

Supplementary Table S1: Hematological Parameters of Infected Control, Malnourished, and Refed Mice, Treated or Untreated.

Groups	Infect control	Infect malnourished	Infect refed	Treated control	Treated malnourished	Treated refed
**RBC (million/mm³)**	9.12 ± 0.09	8.98 ± 0.53	8.75 ± 0.37	9.08 ± 0.10	9.70 ± 0.30	8.67 ± 0.55
**Hemoglobin (g/dL)**	13.35 ± 0.21	12.59 ± 0.66	12.50 ± 0.68	13.10 ± 0.26	13.75 ± 0.49	12.67 ± 0.55
**Hematócrit (%)**	45.05 ± 0.64	42.23 ± 2.58	43.82 ± 1.8	44.13 ± 0.86	46.05 ± 0.07	43.40 ± 2.81
**MCV (fm³)**	49.60 ± 1.41	42.71 ± 11.39	50.10 ± 0.66	48.60 ± 0.62	47.5 ± 1.41	50.07 ± 0.25
**MCH (pg)**	14.65 ± 0.07	14.03 ± 0.26	14.28 ± 0.23	14.43 ± 0.15	13.65 ± 0.78	14.63 ± 0.32
**MCHC (g/dL)**	29.55 ± 1.06	29.84 ± 0.56	28.52 ± 0.54	29.67 ± 0.51	29.85 ± 1.06	29.2 ± 0.66
**Leucocyte (million/mm³)**	3.05 ± 0.35	1.33 ± 0.40*	2.63 ± 1.02 #	4.45 ± 0.99	2.1 ± 0.28*	2.93 ± 1.50
**Platelets (million/mm³)**	843.5 ± 199.40	943.86 ± 157.98	1074 ± 190.69	648.33 ± 75.59	889 ± 158.39	653.33 ± 442.65

At the conclusion of the experiment, BALB/c mice were anesthetized, and blood samples were collected for hematological analysis. The results are the mean ± standard error from three independent experiments. The following abbreviations are used: RBC – red blood cells; MCV – mean corpuscular volume; MCH – mean corpuscular hemoglobin; MCHC – mean corpuscular hemoglobin concentration. Statistical significance as indicated: * $p \leq 0.05$ (Malnourished vs. Control) and # $p \leq 0.05$ (Refed vs. Malnourished).

Supplementary Table S2: Biochemical Parameters of Infected Control, Malnourished, and Refed Mice, Treated or Untreated.

Groups	Infect control	Infect malnourished	Infect refed	Treated control	Treated Malnourished	Treated refed
**Sodium (mEq/L)**	151.67 ± 1.53	155.67 ± 2.52	145.8 ± 5.02	150.33 ± 2.08	152 ± 3.54	151.25 ± 2.5
**Potassium (mEq/L)**	3.13 ± 0.15	3.77 ± 0.59	2.56 ± 0.30	2.90 ± 0.17	2.75 ± 0.49	2.85 ± 0.26
**Glucose (mg/dL)**	109 ± 4.58	78.33 ± 6.66	268.8 ± 37.33 ++++	105.33 ± 11.68	137.5 ± 7.78	211.88 ± 33.41++
**Urea (mg/dL)**	42.43 ± 1.6	46.13 ± 2.53	33.58 ± 2.74	42.93 ± 4.25	39.65 ± 3.18	31.03 ± 2.07
**Albumin (g/dL)**	1.97 ± 0.06	1.97 ± 0.12	1.98 ± 0.13	1.93 ± 0.06	2.15 ± 0.07	1.9 ± 0.16
**Calcium (mg/dL)**	10.3 ± 0.53	9.83 ± 0.35	10.05 ± 0.83	10.27 ± 0.21	10.35 ± 0.21	9.9 ± 0.28
**AST (U/L)**	115.33 ± 16.77	330 ± 0.01	196.80 ± 103.24	101.67 ± 24.58	140 ± 53.74	127 ± 0.01
**ALT (U/L)**	60.33 ± 6.51	83.67 ± 16.26	140.6 ± 40.02	51 ± 2.65	47 ± 4.24	78.67 ± 22.74
**Creatine Kinase (U/L)**	432 ± 15.56	1254 ± 250.1	522.5 ± 79.61	398 ± 209.30	568.5 ± 286.38	502 ± 194.12
**Alkaline phosphatase (U/L)**	135.67 ± 30.29	93 ± 0.01	151.8 ± 16.43	88.67 ± 6.43	76 ± 1.41	82 ± 2.83
**Cholesterol (mg/dL)**	65.50 ± 23.33	84.25 ± 8.89*	71.6 ± 6.73	57 ± 8.0	103.5 ± 9.19*	66.5 ± 11.68
**Iron mg/dL)**	109.67 ± 54.24	86 ± 0.01	98 ± 22.06	149 ± 11.53	61.5 ± 6.36	108 ± 29.7
**Total proteins (g/dL)**	4.27 ± 0.12	4.17 ± 0.12	4.26 ± 0.13	4.5 ± 0.10	4.65 ± 0.07	4.35 ± 0.24

At the end of the experiment, BALB/c mice were anesthetized, and blood samples were collected for biochemical analysis. The results are the mean ± standard error from three independent experiments. Statistical significance was indicated: * $p \leq 0.05$; ++ $p \leq 0.009$; ++++ $p < 0.0001$. (*) indicates comparisons between Malnourished and Control groups; (+) indicates comparisons between Refed and Control groups.
